# One-year outcomes of the RESILIA balloon-expandable transcatheter valve: survival, hemodynamics, and hypoattenuated leaflet thickening

**DOI:** 10.1007/s12928-025-01227-1

**Published:** 2025-12-15

**Authors:** Kazuki Suruga, Vivek Patel, Yuchao Guo, Hidemasa Shitan, Daniel Ng, Takashi Nagasaka, Jaideep Menda, Adishwar Singh, Mitch Gheorghiu, Dhairya Patel, Aakriti Gupta, Tarun Chakravarty, Wen Cheng, Yuito Okada, Hasan Jilaihawi, Mamoo Nakamura, Raj R. Makkar

**Affiliations:** 1 Cedars-Sinai Medical Center, Smidt Heart Institute, 127 S. San Vicente Boulevard, Advanced Health Sciences Pavilion, Third Floor, Suite A3100, Los Angeles, CA 90048 USA; 2 Department of Cardiovascular Medicine, Dentistry and Pharmaceutical Sciences, Okayama University Graduate School of Medicine, Okayama, Japan; 3 Department of Cardiology of The Second Affiliated Hospital, School of Medicine, Zhejiang University, Hangzhou, China; 4 University of California, Los Angeles, CA USA; 5 Department of Cardiovascular Medicine, Gunma University Graduate School of Medicine, Gunma, Japan; 6 Cancer Epidemiology Program, University of Hawaii Cancer Center, Honolulu, HI USA

**Keywords:** **S**APIEN 3 Ultra RESILIA, Transcatheter aortic valve replacement, Hypoattenuated leaflet thickening

## Abstract

Performance evaluation of the latest fifth-generation SAPIEN 3 Ultra RESILIA (S3UR) valve in patients who underwent transcatheter aortic valve replacement (TAVR) is limited. The aim of this study was to compare the 1-year clinical outcomes, including valve performance, in patients undergoing TAVR with the S3UR compared to the SAPIEN 3 or SAPIEN 3 Ultra (S3/S3U). Patients with severe native aortic stenosis who underwent TAVR with either the S3UR or the S3/S3U valve were matched using propensity scores. Predictors of 1-year outcomes were identified. A subgroup analysis of patients who underwent post-TAVR CT was conducted. Among 4,908 patients, 312 matched pairs were assessed. At 1-year, compared to the S3/S3U group, the S3UR group exhibited lower all-cause mortality (HR: 0.42; 95% CI: 0.18–0.96; p = 0.041). The S3UR group demonstrated superior hemodynamic performance, including lower transvalvular gradients, larger effective orifice areas, and decreased incidence of severe prosthesis-patient mismatch and paravalvular leak. In patients who underwent post-TAVR CT (n = 280), prevalence of hypoattenuated leaflet thickening (HALT) at 30-day was lower in the S3UR group compared to S3/S3U (5.7% vs 16.2%; p = 0.006). In multivariate analysis, the S3UR group was independently associated with lower 1-year mortality and reduced HALT. In this propensity score-matched cohort, compared with the S3/S3U group, the S3UR group demonstrated favorable clinical outcomes and superior hemodynamic performance. In subgroup analysis, S3UR was also associated with a lower HALT incidence.

**Graphical abstract:**

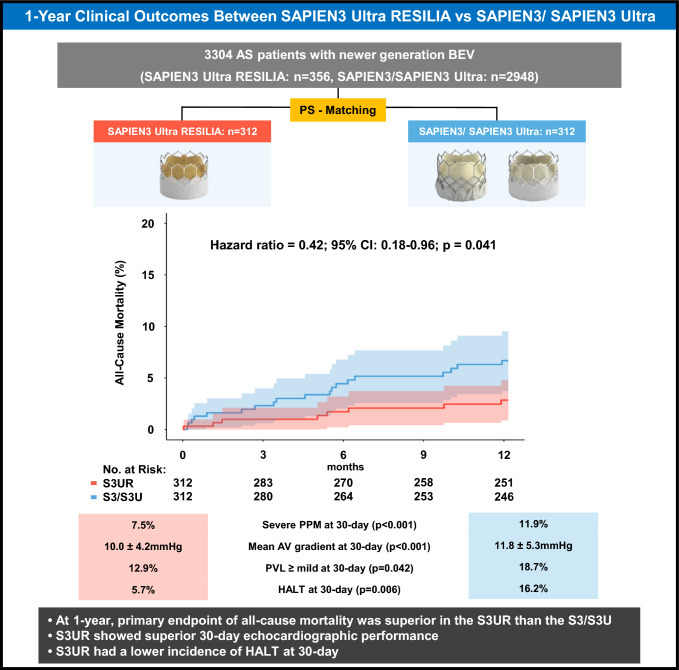

**Supplementary Information:**

The online version contains supplementary material available at 10.1007/s12928-025-01227-1.

## Introduction

The Edwards SAPIEN series (Edwards Lifesciences, Irvine, CA, USA) is the most widely utilized balloon-expandable valve (BEV) platform. The latest fifth-generation SAPIEN 3 Ultra RESILIA (S3UR) incorporates structural modifications, along with innovative features such as dry tissue storage and RESILIA anticalcification technology, which prevent calcium deposition on the valve tissue. Recent studies have demonstrated superior 1-year clinical outcomes and hemodynamic performance with S3UR, including lower gradients, larger effective orifice areas (EOA), and reduced paravalvular leak (PVL), compared to its predecessors [1, 2]. However, these findings are derived from large registries (STS/TVT and OCEAN-TAVI), which lack detailed patient-specific pre-procedural anatomical characteristics obtained by computed tomography (CT) scan. Specifically, precise anatomical features such as annulus size, aortic angle, coronary height, aortic valve calcium burden, and the presence or absence of left ventricular outflow tract (LVOT) calcium, all of which potentially influence valve performance, were not fully captured. Additionally, while RESILIA tissue technology has shown promise in surgical aortic valves [3, 4], its specific impact in transcatheter aortic valve replacement (TAVR) remains limited.

Utilizing propensity score matching, from a large, single-center, real-world population, we aimed to evaluate the 1-year clinical outcomes as well as valve performance, including the incidence of leaflet thrombosis, in patients who underwent TAVR with the S3UR valve compared to the earlier-generation SAPIEN 3 (S3) and SAPIEN 3 Ultra (S3U) valves.

## Material and methods

### Study design and population

This single-center, retrospective, observational study included patients with severe aortic stenosis (AS) who underwent TAVR using a BEV (S3, S3U or S3UR) (Edwards Lifesciences, Irvine, CA, USA). From June 2015 to March 2024, a total of 4908 patients underwent TAVR in our institution. Patients who underwent valve-in-valve procedures, had pure aortic regurgitation, received valves other than the aforementioned three BEVs, had incomplete pre-procedural CT data, or had unsuitable CT image quality (e.g., non-contrast CT) were excluded. Pre-procedural CT imaging was performed using a second-generation dual-source CT system (Siemens Somatom Definition Flash; Siemens Healthcare, Erlangen, Germany), as previously described [5]. A multidisciplinary heart team assessed all patients for TAVR eligibility. The choice and sizing of the transcatheter heart valve (THV) followed labeled indications with reference to the annulus area measured by CT and transesophageal echocardiography, with the final decision made by the heart team.

The study was approved by the Institutional Review Board of Cedars-Sinai Medical Center, Los Angeles, CA (IRB Protocol ID: STUDY00003568). This study adhered to the ethical standards of the Declaration of Helsinki (1975) and informed consent was obtained from all patients. Data on medical history and comorbidities were obtained from medical records. Transthoracic echocardiography (TTE) was performed at baseline, at discharge, at 30-day, and at 1-year. Additionally, assessment of the New York Heart Association (NYHA) classification, and Kansas City Cardiomyopathy Questionnaire–Overall Summary score (KCCQ-OS) was conducted at baseline, at 30-day and at 1-year.

To detect leaflet thrombosis and evaluate prosthetic valve geometry, CT examinations were repeated 30-day post-TAVR implantation as part of the RESOLVE (Assessment of Transcatheter and Surgical Aortic Bioprosthetic Valve Thrombosis and Its Treatment with Anticoagulation; NCT02318342) registry. The indication for CT was not strictly protocol-driven; imaging was performed at variable time points after TAVR, either as part of routine follow-up (typically around 30 days post-procedure) or when patients with prior valve implantation presented for clinically indicated follow-up during the registry period. CT analyses were performed using a standardized four-dimensional, volume-rendered imaging protocol. All scans were independently evaluated by readers blinded to procedural details, valve type, and clinical outcomes at the core laboratory of Cedars-Sinai Medical Center [6].

### Endpoints

The primary endpoint of the study was all-cause mortality at 1-year following TAVR. The secondary endpoints were echocardiographic and functional outcomes. In the subgroup analysis, the incidence of leaflet thrombosis was evaluated in patients who underwent follow-up CT imaging at 30-day after TAVR.

### Definitions

Clinical outcomes were defined according to the latest Valve Academic Research Consortium 3 (VARC-3) definitions. Prosthesis-patient mismatch (PPM) was assessed according to the VARC-3 definition from the indexed effective orifice area (iEOA) [7]. Moderate PPM was defined by iEOA >0.65 and ≤ 0.85 cm^2^/m^2^ (>0.55 and ≤ 0.70 cm^2^/m^2^ if BMI ≥ 30kg/m^2^) and severe PPM by iEOA ≤ 0.65 cm^2^/m^2^ (≤ 0.55cm^2^/m^2^ if BMI ≥ 30 kg/m^2^). For PPM assessment, the EOA at 30-day was measured using TTE and calculated via the continuity equation. Aortic valve reintervention was defined as any of the following: valve-in-valve TAVR, balloon dilatation, paravalvular leak closure, or surgical revision.

### Pre- and post-procedural CT analysis

#### Pre-procedural CT assessment

The extent of calcification was evaluated at the LVOT, annulus, and sinotubular junction (STJ) and the aortic valve calcium volume was measured using a dedicated tool within the 3mensio (3mensio Medical Imaging), as we previously reported [5]. Calcification was graded using a semiquantitative system as follows: grade 0, no calcification; grade 1, presence of a calcium nodule < 5 mm in any dimension and covering < 10% of the LVOT, annular, or STJ perimeter; grade 2, presence of 2 nodules or a single nodule >5 mm in any dimension or covering >10% of the LVOT, annular, or STJ perimeter; and grade 3, presence of multiple nodules merging into a focus >1 cm in any dimension or covering >20% of the LVOT, annular, or STJ perimeter [5].

The LVOT was measured from a perpendicular plane within the left ventricle positioned 4 mm below the annulus plane. Aortic angulation was calculated from the coronal view at the annular level and defined as the angle subtended by the annular plane and horizontal reference.

#### Post-procedural CT assessment

Hypoattenuated leaflet thickening (HALT) was defined as a discernible increase in leaflet thickness in the diastolic phase (Figure S1A, B). If HALT was identified, a meticulous assessment of leaflet motion was conducted using 4-dimensional CT imaging [8]. Motion reduction of each leaflet was evaluated using multiphase volume-rendered enface cine projection. Hypoattenuation affecting motion (HAM) was defined as >50% reduction in leaflet motion relative to the radius of the bioprosthetic frame (Figure S1C). To quantify the geometry of the THV post-TAVR, we incorporated four CT-derived parameters—post-implant oversizing, THV expansion, eccentricity, and prosthesis deformation index—based on the methodology described by Fukui et al [9]. All measurements were obtained at specific anatomical levels, including the frame inflow, native annulus, prosthesis waist, and leaflet inflow (Figure S1D, E).

### Statistical analyses

Continuous variables were presented as mean ± standard deviation, or median (inter-quartile range [IQR]), depending on the distribution of the data, whereas categorical variables were presented as number (percentage). Mann–Whitney *U*, Student’s t-test, or Chi-square test was used for comparison between the groups for continuous and categorical variables, as appropriate. Time-to-event outcomes, including all-cause mortality, cardiac mortality, heart failure (HF) rehospitalization, disabling stroke, aortic valve reintervention, myocardial infarction, and life-threatening bleeding, were analyzed using the Kaplan-Meier method with the Cox proportional hazards model. Given the nonrandomized design of the study, propensity score matching was used to adjust for any confounding variables, taking into consideration clinical characteristics. The propensity score matching was calculated using a logistic regression model based on 24 relevant variables that may affect annulus size as well as study outcomes (Table S1). A 1:1 propensity score matching was performed using the nearest neighbor method with a caliper width set at 0.2 standard deviations (SDs) of the propensity score logit to ensure closely matched pairs. To ensure the effectiveness of the matching, standardized mean difference (SMD) was used to assess and compare the balance of covariates between the treatment groups after propensity score matching, with a value of < 10% indicating a good balance. Sensitivity analyses using 1:2 and 1:3 matching yielded results consistent with the primary 1:1 analysis. Due to <2% missingness in baseline information, propensity score matching with weighting was performed using complete-case analysis. Additionally, Cox proportional hazards models were used to assess 1-year mortality. To explore whether residual PVL contributed to mortality differences between valve types, we constructed two sequential multivariable models: Model 1 with device type and baseline covariates, and Model 2 with additional adjustment for mild or greater PVL mild at discharge. Statistical significance was set at p < 0.05. All statistical analyses were conducted using SPSS software (version 24.0; IBM Corporation, Armonk, NY, USA).

## Results

### Study population and baseline characteristics

Between August 2015 and March 2024, this study included 3304 consecutive patients who underwent TAVR for native AS. A total of 356 S3UR patients were 1:1 propensity score-matched with 2948 S3/S3U patients in this study, resulting in 312 patients in each group (Figure S2). Table 1 shows the baseline characteristics of both unmatched and matched cohorts. In this matched cohort, patients with small annulus were well balanced compared to those with large annulus with SMD < 0.10 across all measured baseline characteristics, except for NYHA functional class III/IV (SMD=0.104). Table 2 summarizes the pre-procedural CT measurements between the two matched groups. Anatomical features including annulus size, sinus of Valsalva, sinotubular junction, coronary artery heights, and valve calcification were not significantly different between groups.

**Table 1:** Baseline characteristics of the unmatched the matched population

		Prematching cohort				Matched cohort			
	Overall (n=3304)	S3UR (n=356)	S3/S3U (n=2948)	Missing (%)	p Value	S3UR (n=312)	S3/S3U (n=312)	p Value	SMD
Age, y	79.1 ± 9.8	74.2 ± 9.9	79.7 ± 9.7	0	<0.001	74.2 ± 9.8	75.1 ± 11.5	0.254	0.094
Male	2509 (62.3)	234 (65.7)	1825 (61.9)	0	0.160	212 (67.9)	206 (66.0)	0.670	0.041
BMI, kg/m^2^	27.0 ± 5.8	26.8 ± 5.6	26.9 ± 5.8	0	0.619	26.5 ± 5.7	26.9 ± 5.6	0.370	0.072
Hypertension	2809 (85.0)	298 (83.7)	2511 (85.2)	0	0.463	255 (81.7)	263 (84.3)	0.456	0.068
Diabetes Mellitus	1051 (31.8)	110 (30.9)	941 (31.9)	0	0.696	98 (31.4)	98 (31.4)	1.000	<0.001
Dyslipidemia	2090 (63.3)	261 (73.3)	1829 (62.0)	0	<0.001	224 (71.8)	227 (72.8)	0.858	0.021
Current smoking	167 (5.1)	15 (4.2)	152 (5.2)	0	0.443	13 (4.2)	14 (4.5)	1.000	0.016
CKD (eGFR<30 mL/min)	326 (9.9)	34 (9.6)	292 (9.9)	0	0.832	25 (8.0)	29 (9.3)	0.669	0.046
STS Mortality Score	4.7 ± 4.7	3.8 ± 4.3	4.8 ± 4.7	0	<0.001	3.7 ± 4.2	3.9 ± 4.4	0.511	0.053
NYHA functional class, III or IV	2662 (80.6)	193 (54.2)	2469 (83.8)	0	<0.001	192 (61.5)	56.4 (56.4)	0.222	0.104
CAD	1399 (42.3)	189 (53.1)	1210 (41.0)	0	<0.001	152 (48.7)	163 (52.2)	0.423	0.071
Prior MI	442 (13.4)	53 (14.9)	389 (13.2)	0	0.376	46 (14.7)	50 (16.0)	0.739	0.036
Prior CABG	339 (10.3)	32 (9.0)	307 (10.4)	0	0.403	22 (7.1)	26 (8.3)	0.652	0.048
Prior PCI	705 (21.3)	77 (21.6)	628 (21.3)	0	0.887	71 (22.8)	70 (22.4)	1.000	0.008
PAD	396 (12.0)	38 (10.7)	358 (12.1)	0	0.420	34 (10.9)	34 (10.9)	1.000	<0.001
AF	920 (27.8)	93 (26.1)	827 (28.1)	0	0.443	93 (29.8)	87 (27.9)	0.659	0.042
Prior Stroke	209 (6.3)	31 (8.7)	178 (6.0)	0	0.051	28 (9.0)	28 (9.0)	1.000	<0.001
COPD	417 (12.6)	51 (14.3)	366 (12.4)	0	0.305	46 (14.7)	45 (14.4)	1.000	0.009
Medications									
Aspirin	1354/2209 (61.3)	176/356 (49.4)	1178/1853 (63.6)	36.7	<0.001	151 (48.4)	164 (52.6)	0.224	0.084
P2Y12 inhibitor	352/2206 (16.0)	35/356 (9.8)	317/1850 (17.1)	36.8	0.001	31 (9.9)	40 (12.8)	0.354	0.091
Any oral anticoagulation	438/2208 (19.8)	65/356 (18.3)	373/1852 (20.1)	43.1	0.415	56 (17.9)	62 (19.9)	0.488	0.049
Echocardiographic data									
LVEF, %	58.2 ± 14.3	57.8 ± 13.5	58.2 ± 14.4	1.8	0.666	57.2 ± 14.6	57.8 ± 13.5	0.572	0.045
AV mean gradient, mmHg	38.5 ± 14.7	36.0 ± 14.1	38.8 ± 14.7	1.2	0.009	37.7 ± 13.6	38.7 ± 14.1	0.376	0.072
EOA, cm^2^	0.80 ± 0.32	0.82 ± 0.28	0.75 ± 0.30	1.5	0.085	0.81 ± 0.30	0.80 ± 0.27	0.498	0.035
AR ≥ moderate	499 (16.0)	63 (17.7)	461 (15.9)	0.3	0.451	52 (16.7)	49 (15.7)	0.804	0.026
MR ≥ moderate	266 (14.0)	17 (8.1)	249 (14.7)	5.6	0.009	14 (4.5)	21 (6.7)	0.882	0.022
TR ≥ moderate	351 (15.3)	20 (9.4)	331 (15.9)	4.3	0.012	17 (5.4)	15 (4.8)	0.392	0.083

Values are n (%) or mean ± SD. AF, atrial fibrillation; AR, aortic regurgitation; AVA, aortic valve area; BMI, body mass index; CAD, coronary artery disease; CABG, coronary artery by-pass graft; CKD, chronic kidney disease; COPD, chronic obstructive pulmonary disease; IQR, inter-quartile range; LVEF, left ventricular ejection fraction; MI, myocardial infarction; MR, mitral regurgitation; NYHA, New York Heart Association; PAD, peripheral artery disease; PCI, percutaneous coronary intervention; PM, pacemaker; SMD, standardized mean difference; STS, society of thoracic surgeons; TR, tricuspid regurgitation.

**Table 2:** Pre-procedural CT analysis in the matched population

	S3UR	S3/S3U	SMD
Pre-procedure	(n=312)	(n=312)	
Bicuspid aortic valve	56 (17.9)	52 (16.7)	0.034
Annulus mean diameter, mm	24.9 ± 2.9	25.0 ± 2.6	0.057
Annulus area, mm^2^	494.5 ± 117.4	483.7 ± 101.4	0.007
Annulus perimeter, mm	79.4 ± 9.4	79.5 ± 8.1	0.012
Mean diameter of Sinus of Valsalva, mm	31.5 ± 4.0	31.2 ± 4.2	0.070
STJ height from annulus, mm	24.6 ± 3.9	24.2 ± 3.8	0.101
STJ mean diameter, mm	29.1 ± 3.9	29.4 ± 3.8	0.063
LVOT mean diameter, mm	25.0 ± 5.6	24.8 ± 4.2	0.034
LVOT area, mm^2^	475.7 ± 128.6	479.9 ± 116.9	0.034
LVOT perimeter, mm	78.2 ± 10.6	77.9 ± 9.8	0.031
LCA height, mm	14.9 ± 3.2	14.8 ± 3.8	0.035
RCA height, mm	18.2 ± 3.2	18.5 ± 3.4	0.081
Calcium distribution			
Annular calcification grade			0.080
0	227 (72.8)	208 (66.7)	
1	60 (19.2)	72 (23.1)	
2	21 (6.7)	29 (9.3)	
3	4 (1.3)	3 (1.0)	
LVOT calcification grade			0.050
0	268 (85.9)	272 (87.2)	
1	31 (9.9)	25 (8.0)	
2	11 (3.5)	15 (4.8)	
3	2 (0.6)	0 (0)	
STJ calcification grade			0.140
0	244 (78.2)	226 (72.4)	
1	53 (17.0)	75 (24.0)	
2	12 (3.8)	11 (3.5)	
3	3 (1.0)	0 (0)	
Calcium volume, mm^3^	289.4 ± 257.0	268.5 ± 226.5	0.081
Aortic angulation, deg	49.5 ± 9.8	48.6 ± 9.5	0.087

Values are n (%) or mean ± SD. AVC, aortic valve calcium; CT, Computed Tomography; LCA, left coronary artery; LVOT, left ventricular outflow tract; RCA, right coronary artery; STJ, sino-tubular junction.

### Procedural and clinical outcomes

Procedural variables, including valve size selection, rates of pre-dilatation and post-dilatation, were comparable between groups. The incidences of annular rupture or aortic dissection, coronary obstruction, and major vascular complications were low and not different between the groups (Table S2).

**Fig 1:**
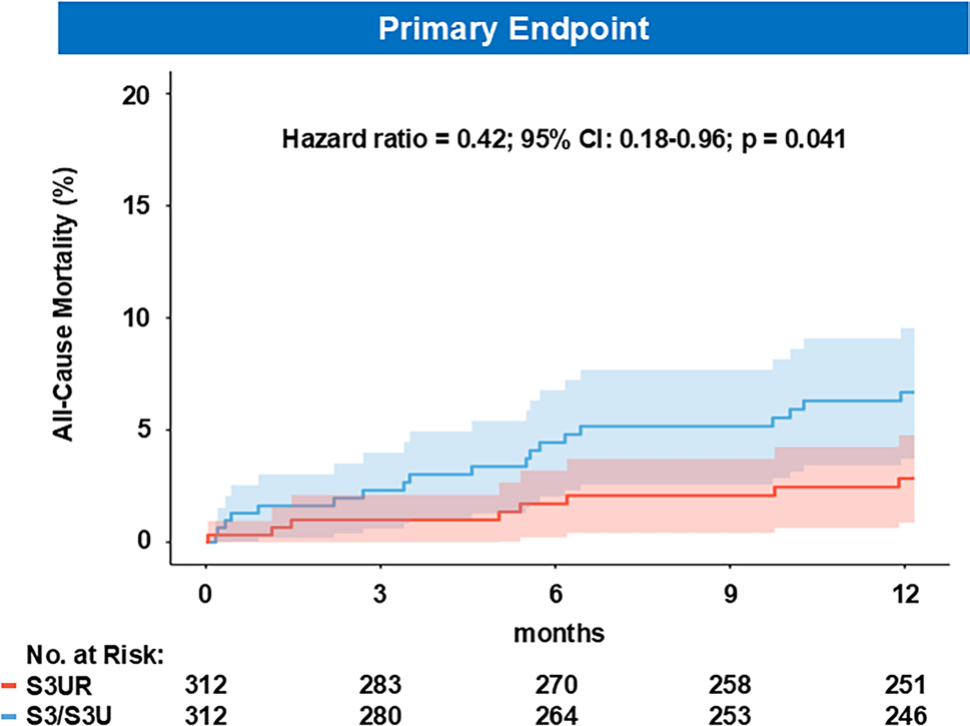
Kaplan-Meier Estimates of Mortality at 1 Year For all-cause mortality, Cox proportional hazards models were used. Shaded areas represent 95% CIs. HR = hazard ratio; S3 = SAPIEN3: S3U = SAPIEN3 Ultra; S3UR = SAPIEN3 Ultra RESILIA

**Fig 2:**
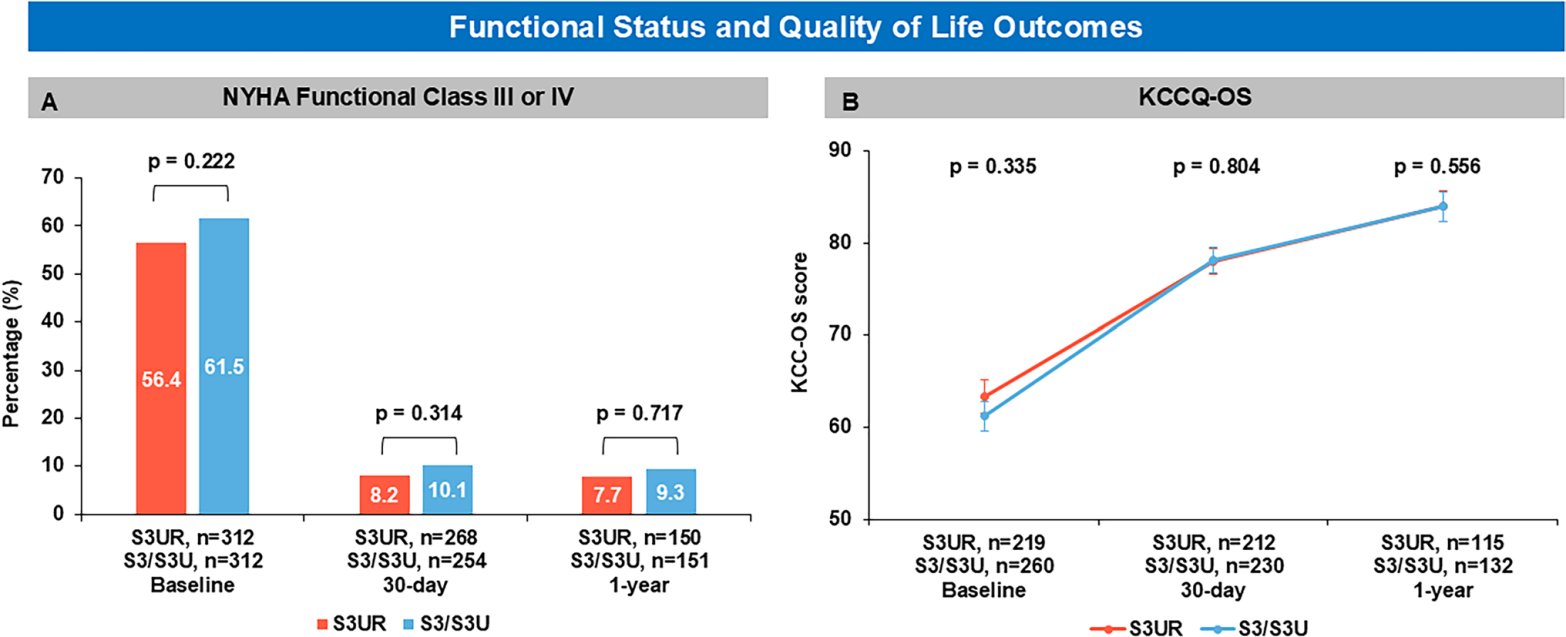
Functional Status and Quality of Life Outcomes. **A** NYHA class III or IV status at baseline, 30-day, and 1-year for SAPIEN3 Ultra RESILIA(S3UR) and SAPIEN3 (S3) or SAPIEN3 Ultra (S3U) patients. **B** KCCQ-OS score at baseline, 30-day, and 1-year. Error bars represent the standard error of the mean. KCCQ-OS, Kansas City Cardiomyopathy Questionnaire Overall Summary Score; NYHA, New York Heart Association

As demonstrated in Table 3, all-cause mortality, cardiac mortality, and stroke at 30-day were low, with no significant differences between the 2 groups. However, 1-year all-cause mortality was lower in the S3UR group (HR: 0.42; 95% CI:0.18–0.96.18.96; p = 0.041) (Fig 1). Aortic valve reintervention remained rare in both groups at 1 year (0.3% vs 0%). Both groups had similar functional improvement after TAVR assessed using both NYHA functional class and KCCQ-OS (Fig 2).

**Table 3:** Clinical outcomes in the matched population

	S3UR (n=312)	S3/S3U (n=312)	OR/HR (95%CI)	p-value
30-day clinical outcomes				
All-cause mortality	1 (0.3)	4 (1.3)	0.25 (0.03–2.22.03.22)	0.214
Cardiac mortality	1 (0.3)	3 (1.0)	-	NR^a^
Disabling stroke	1 (0.3)	1 (0.3)	1.00 (0.06–16.11.06.11)	1.00
HF rehospitalization	2 (0.6)	0 (0.0)	-	NR^a^
New permanent pacemaker implantation	20 (6.4)	17 (5.4)	1.18 (0.63–2.20.63.20)	0.612
Life-threatening bleeding	4 (1.3)	1 (1.3)	4.00 (0.45–35.59.45.59)	0.214
1-year clinical outcomes				
All-cause mortality	8 (2.6)	19 (6.1)	0.42 (0.18–0.96.18.96)	0.041
Cardiac mortality	3 (1.0)	7 (2.2)	0.28 (0.06–1.36.06.36)	0.114
Disabling stroke	3 (1.0)	4 (1.3)	0.68 (0.08–5.62.08.62)	0.718
HF rehospitalization	5 (1.6)	6 (1.9)	0.81 (0.28–2.37.28.37)	0.698
Myocardial Infarction	3 (1.0)	1 (0.3)	1.21 (0.27–5.40.27.40)	0.805
Life-threatening bleeding	7 (2.2)	4 (1.3)	1.96 (0.58–6.62.58.62)	0.278
Aortic valve reintervention	1 (0.3)	0 (0)	-	NR^a^

Values are Kaplan-Meier estimate % (n events). ^a^NR because of inaccuracy in zero event rate comparisons. OR, Odds Ratio; HR, Hazard Ratio; CI, confidence intervals; HF, heart failure; NR=not reported; other abbreviations as in Table 1.

Determinants of all-cause mortality were evaluated using multivariable regression analysis (Table 4). In Model 1, which included device type and baseline clinical covariates, S3UR remained independently associated with reduced 1-year all-cause mortality (HR 0.32; 95% CI 0.13–0.80; p = 0.015). In Model 2, after additional adjustment for discharge mild or greater PVL, PVL emerged as a strong predictor of mortality (HR 6.66; 95% CI 1.43–31.08; p = 0.016), while the association between S3UR and mortality became weaker and was no longer statistically significant (HR 0.66; 95% CI 0.17–2.58; p = 0.55).

**Table 4:** Multivariable Cox model for probability of 1-year all-cause mortality

Variables	Univariate		Model-1		Model-2	
	HR (95%CI)	p-value	HR (95%CI)	p-value	HR (95%CI)	p-value
Age	0.99 (0.95–1.03.95.03)	0.608				
Male	0.86 (0.40–1.87.40.87)	0.701				
BMI (kg/m^2^)	1.01 (0.93–1.11.93.11)	0.742				
Diabetes Mellitus	2.12 (0.99–4.52.99.52)	0.051				
Atrial fibrillation	2.48 (1.19–5.19.19.19)	0.016	2.10 (0.91–4.81.91.81)	0.081	2.12 (0.55–8.21.55.21)	0.280
Prior stroke	2.47 (0.93–6.60.93.60)	0.071				
Chronic lung disease	1.41 (0.54–3.73.54.73)	0.485				
Coronary artery disease	1.10 (0.52–2.31.52.31)	0.812				
STS score (per 1% increase)	1.08 (1.04–1.12.04.12)	<0.001	1.05 (1.00–1.11.00.11)	0.036	1.01 (0.94–1.08.94.08)	0.830
NYHA functional class, III or IV	4.37 (1.53–12.40.53.40)	0.006	4.36 (1.28–14.86.28.86)	0.019	2.05 (0.51–8.21.51.21)	0.310
LVEF (%)	0.97 (0.95–0.99.95.99)	0.021				
MR ≥ moderate at baseline	2.24 (0.80–6.28.80.28)	0.125				
TR ≥ moderate at baseline	3.09 (0.99–9.58.99.58)	0.051				
PVL ≥ mild (at discharge)	5.33 (1.35–21.00.35.00)	0.017	-	-	6.66 (1.43–31.08.43.08)	0.016
PPM (>moderate) (at 30-day)	1.44 (0.29–7.14.29.14)	0.658				
Annular calcium grade moderate or greater	1.04 (0.13–8.14.13.14)	0.968				
Life-threatening bleeding (In-hospital)	23.20 (4.66–115.00.66.00)	<0.001	20.52 (5.09–82.68.09.68)	<0.001	23.37 (3.05–179.18.05.18)	0.002
S3UR vs S3/S3U	0.42 (0.18–0.96.18.96)	0.041	0.32 (0.13–0.80.13.80)	0.015	0.66 (0.17–2.58.17.58)	0.550

Data are presented as an estimate (95% confidence interval)

HR = hazard ratio; CI = confidence interval; other abbreviations as in Table 1.

### Echocardiographic outcomes

Table 5 presents hemodynamic outcomes from discharge through 1-year follow-up. At discharge, patients in the S3UR group exhibited significantly lower mean gradients (9.0 ± 4.3 mmHg vs. 11.7 ± 4.8 mmHg; p < 0.001) and larger effective orifice areas (EOA) (1.87 ± 0.49 cm^2^ vs. 1.66 ± 0.38 cm^2^; p < 0.001) compared to those in the S3/S3U group. These trends were sustained at 30-day and at 1-year. Additionally, the S3UR group demonstrated a significantly lower prevalence of severe PPM at 30-day (7.5% vs. 11.9%; p < 0.001). The incidence of mild or greater PVL was also consistently lower in the S3UR group at discharge, 30-day, and persisted through 1-year. When comparing the devices individually, the incidence of mild or greater PVL was comparable between the S3UR and S3U group (Figure S3).

**Table 5:** Discharge, 30-day, and 1-year echocardiographic outcomes in the matched cohort

	S3UR	S3/S3U	p value
Discharge	(n=312)	(n=312)	
LVEF, %	61.2 ± 13.4	63.1 ± 11.7	0.231
Aortic mean gradient, mm Hg	9.0 ± 4.3	11.7 ± 4.8	<0.001
Aortic mean gradient ≥20 mmHg	8 (2.6)	15 (4.8)	0.311
EOA, cm^2^	1.87 ± 0.49	1.66 ± 0.38	<0.001
Paravalvular regurgitation			0.006
Non/trace	294 (94.2)	267 (85.6)	
Mild	15 (4.8)	41 (13.1)	
Mild-to-moderate	3 (1.0)	4 (1.3)	
Moderate	0 (0)	0 (0)	
Mild or greater	18 (5.8)	45 (14.4)	<0.001
MR ≥ moderate	17 (5.4)	16 (5.1)	0.858
TR ≥ moderate	20 (6.4)	32 (10.3)	0.128
30-day device performance	(n=240)	(n=252)	
LVEF, %	58.8 ± 12.1	60.0 ± 11.8	0.279
Aortic mean gradient, mm Hg	10.0 ± 4.2	11.8 ± 5.3	<0.001
Aortic mean gradient ≥20 mmHg	11 (4.6)	14 (5.6)	0.545
EOA, cm^2^	1.71 ± 0.56	1.47 ± 0.43	<0.001
Severe measured PPM^a^	18 (7.5)	30 (11.9)	<0.001
Paravalvular regurgitation			0.073
Non/trace	209 (87.1)	205 (81.3)	
Mild	28 (11.7)	41 (16.3)	
Mild-to-moderate	3 (1.2)	5 (2.0)	
Moderate	0 (0)	1 (0.4)	
Mild or greater	31 (12.9)	47 (18.7)	0.042
MR ≥ moderate	13 (5.4)	15 (6.0)	0.572
TR ≥ moderate	9 (3.8)	18 (7.1)	0.194
1-year device performance	(n=138)	(n=170)	
LVEF, %	58.9 ± 11.1	60.4 ± 11.4	0.277
Aortic mean gradient, mm Hg	10.3 ± 5.0	12.9 ± 6.9	<0.001
Aortic mean gradient ≥20 mmHg	4 (2.9)	14 (8.2)	0.031
EOA, cm^2^	1.71 ± 0.57	1.53 ± 0.42	0.035
Paravalvular regurgitation			0.108
Non/trace	120 (87.0)	134 (78.8)	
Mild	16 (11.6)	28 (16.5)	
Mild-to-moderate	2 (1.4)	5 (2.9)	
Moderate	0 (0)	3 (1.8)	
Mild or greater	18 (13.0)	36 (21.2)	0.038
MR ≥ moderate	8 (5.8)	9 (5.3)	0.862
TR ≥ moderate	9 (6.5)	14 (8.2)	0.538

Data are presented as an estimate (95% confidence interval)

Abbreviations as in Table 1.

### Subgroup analysis

**Fig 3:**
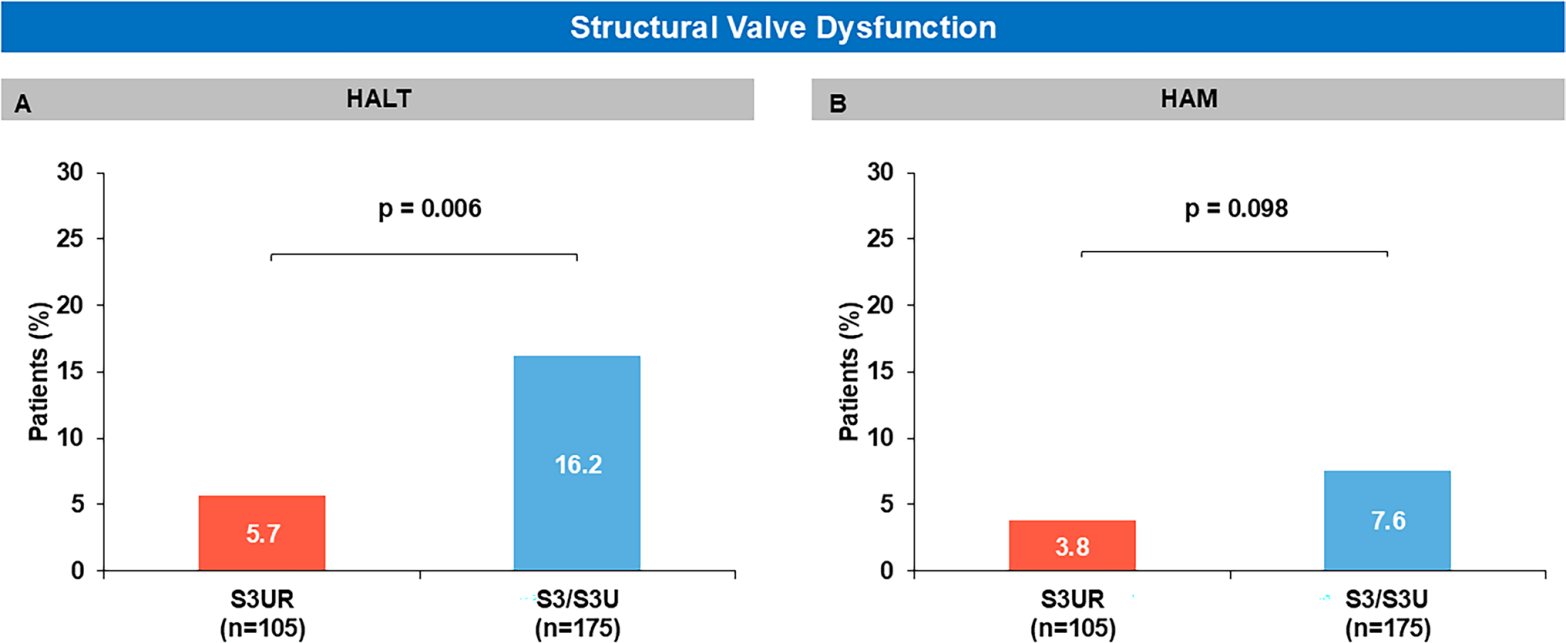
Structural Valve Dysfunction Structural valve dysfunction and its components (HALT and HAM) at 30-day post-procedure. HALT, hypoattenuated leaflet thickening; HAM, hypoattenuation affecting motion; S3 = SAPIEN3: S3U = SAPIEN3 Ultra; S3UR = SAPIEN3 Ultra RESILIA

To examine the effect of valve type on post-procedural valve geometry and subclinical leaflet thrombosis, we performed a predefined CT sub-analysis in 280 patients who underwent 30-day post-TAVR CT. There were no significant differences in THV frame geometry between the two groups, including post-implant oversizing, THV expansion, eccentricity, and prosthesis deformation index (Table S3). At 30-day, the prevalence of HALT was significantly lower in the S3UR group than in the S3/S3U group (HALT: 5.7% vs 16.0%, p = 0.006) (Fig 3). When the three valve types were evaluated separately, HALT was observed in 16.7% of patients treated with S3, 14.5% with S3U, and 5.7% with S3UR (Figure S4). There were no significant differences in HAM, although a similar trend was noted (3.8% vs 7.6%, p = 0.098) (Fig 3). Both univariate and multivariate analyses demonstrated less HALT at 30-day after TAVR in S3UR group compared to S3/S3U group (Table S4).

## Discussion

This study suggests several clinical advantages of the S3UR over the S3/S3U. These are as follows: 1) TAVR with the S3UR is associated with lower 1-year all-cause mortality than the S3/S3U, likely due to less PVL; 2) the prevalence of HALT was significantly lower in the S3UR group than in the S3/S3U group and identified S3UR as an independent predictor of HALT; 3) S3UR demonstrated superior hemodynamic performance, including lower gradients, larger EOA, reduced PVL, and a lower incidence of severe PPM.

The mortality benefit observed with the S3UR in our cohort aligns with findings from the TVT Registry, which attributed improved survival to reduced PVL and fewer life-threatening bleeding events compared to its predecessors [1]. In our study, the separation of event curves appeared earlier than in the TVT Registry. This difference was driven primarily by more favorable early outcomes in the S3UR group rather than inferior performance of the S3/S3U valves. Several center-specific factors may explain this difference. The S3UR’s extended outer skirt and larger EOA enabled operators to avoid aggressive dilatation, likely reducing procedural risk. In addition, a learning-curve effect may have further amplified the intrinsic advantages of S3UR, whereas such center-specific effects are less apparent in large multicenter registries.

To further explore the mechanistic basis of the survival benefit, we performed a sequential Cox analysis. S3UR use was associated with a markedly lower prevalence of mild or greater PVL at discharge, and mild or greater PVL remained an independent predictor of 1-year mortality. Adjustment for PVL reduced the association between S3UR and mortality, suggesting that reduced residual PVL may partially contributes to the observed survival benefit. Although the impact of mild PVL remains controversial and has traditionally been considered clinically acceptable after TAVR, emerging evidence suggests that even mild PVL may be associated with an increased risk of mortality after TAVR [10]. As moderate or severe PVL at discharge with contemporary TAVR devices has declined over the years and was absent in both device groups in our study, the impact of mild PVL on outcomes may have become more pronounced. Long-term implications of mild PVL on patient outcomes warrant further investigation.

In contrast to prior registry findings, life-threatening bleeding did not differ between valve types in our cohort. This may be due to further detailed propensity score matching of the clinical factors including the use of anticoagulation therapy that directly influence bleeding risk, which was comparable between the two groups.

As reported previously, the RESILIA design and its associated improved valve opening are considered as the major contributing factors of larger EOA and reduced incidence of measured severe PPM [1, 2]. However, the clinical impact of severe PPM after TAVR with balloon-expandable devices remain controversial [11]. In the current study, reduction of severe PPM in the S3UR group was not a predictor of adverse outcomes at 1-year. However, as reported recently, the severe PPM may have a greater impact in young and low-risk patients who have higher physiological demands and less comorbidity [12]. Furthermore, Levesque et al. demonstrated that PPM had a negative long-term impact on mortality after TAVR only beyond 4-year follow-up [13]. Long-term follow-up, specifically in young and low-risk patients will be crucial to evaluate the true impact of PPM reduction using the S3UR.

An additional key finding of this study is the lower prevalence of HALT in the S3UR group compared to the S3/S3U group. Prior studies reported that HALT was observed in 13% to 21% of patients who were treated with S3 valves at 30-day [9, 14]. In this study, HALT was observed in 16.2% of patients in the S3/S3U group, while it was only seen in 5.7% of the patients in the S3UR group. As the clinical risk factors of HALT such as increased age, obesity, low left ventricular ejection fraction, absence of anticoagulation, and valve deformation [6, 9, 15], were comparative in this study, and the incidence of HALT was similar between S3 and S3U, the observed HALT reduction in the S3UR group in this study could be attributable to potentially protective differences in leaflet architecture and tissue of the S3UR valve in comparison to its predecessors. To the best of our knowledge, this is the first clinical study to demonstrate lower incidence of HALT of the RESILIA tissue valve (S3UR) compared with conventional S3/S3U valves. Hein et al. reported an association between HALT and symptomatic hemodynamic valve deterioration [16] and therefore, these findings suggest the potential for improved long-term valve durability with this cutting-edge technology.

Finally, the detailed CT analyses performed in this study provide additional mechanistic insight into how S3UR design features contribute to improved hemodynamics and reduced HALT, even in the absence of differences in expansion or frame deformation. These findings extend prior registry observations and further clarify the structural and functional advantages of the S3UR valve.

## Study limitations

This study has some limitations. First, it was a single-center study with a retrospective design, highlighting the need for multi-center studies to validate. Second, although post-TAVR CT imaging was performed in a substantial proportion of patients, it was limited to 44.4% of the cohort, potentially introducing selection bias in the HALT analysis. Third, as we performed propensity score matching, the results may be generalizable only to populations with characteristics similar to those of the matched cohort. Finally, the study follow-up period was limited to 1 year. Longer term studies will be required to determine long-term outcomes.

## Conclusions

In this single-center, propensity score-matched study, the S3UR transcatheter heart valve demonstrated superior 1-year mortality and hemodynamic performance. Additionally, S3UR reduced HALT incidence at 30-day compared to S3/S3U. Further large-scale, multi-center, and longer follow-up will determine the durability and precise clinical impact of the RESILIA technology on the SAPIEN valve platform. 

## Supplementary Information

Below is the link to the electronic supplementary material.

Supplementary file1 (DOCX 1276 KB)

## Data Availability

The deidentified participant data will not be shared.

## Abbreviations

AS: Aortic Stenosis
BEV: Balloon-Expandable Valve
EOA: Effective Orifice Area
HALT: Hypoattenuated Leaflet Thickening
HAM: Hypoattenuation Affecting Motion
PPM: Prosthesis-Patient Mismatch
PVL: Paravalvular Leak
S3: SAPIEN 3
S3U: SAPIEN 3 Ultra
S3UR: SAPIEN 3 Ultra RESILIA
TAVR: Transcatheter Aortic Valve Replacement
