# Supplementary material for: One-year outcomes of the RESILIA balloon-expandable transcatheter valve: survival, hemodynamics, and hypoattenuated leaflet thickening
Source: Cardiovasc Interv Ther. 2025 Dec 15;41(2):414–24. doi: 10.1007/s12928-025-01227-1 (PMC13002764; doi:10.1007/s12928-025-01227-1)

**Supplemental Appendix**

**List**

| **Table S1.** Baseline and procedural characteristics used for propensity score matching. | Page 2 |
| --- | --- |
| **Table S2.** Procedural characteristics and Complications in the Matched Population. | Page 3 |
| **Table S3.** Post-procedural CT analysis in the Sub-group Population | Page 4 |
| **Table S4.** Predictors of hypoattenuated leaflet thickening (HALT) | Page 5 |
| **Figure S1.** Evaluation of hypoattenuated leaflet thickening (HALT), hypoattenuation affecting motion (HAM), and prosthetic valve geometry. | Page 6 |
| **Figure S2.** Study flow chart | Page 7 |
| **Figure S3.** Distribution of 30-Day PVL Severity for S3, S3U, and S3UR Valves | Page 8 |
| **Figure S4.** Incidence of HALT for S3, S3U, and S3UR Valves | Page 9 |

**Table S1.** Baseline and procedural characteristics used for propensity score matching.

|  |  |
| --- | --- |
| Age* | Prior stroke |
| Male sex | Prior coronary artery bypass grafting |
| Body mass index | Peripheral vascular disease |
| Hypertension | Prior percutaneous coronary intervention |
| Dyslipidemia | Coronary artery disease |
| New York Heart Association class (III/IV vs. I/II) | Chronic lung disease |
| STS-PROM score* | Atrial fibrillation/flutter |
| Diabetes mellitus | Bicuspid aortic valve morphology |
| Chronic kidney disease (CKD) (defined as estimated glomerular filtration rate < 30 mL/min/1.73 m2) |  |
| Curren smoker |  |
| Left ventricular ejection fraction* |  |
| Aortic valve mean gradient* |  |
| Sapien valve size (20-29 mm) |  |
| Annular area* |  |
| LVOT area* |  |
| Mean diameter of Sinus of Valsalva * |  |

*As a continuous variable. STS PROM = Society of Thoracic Surgeons Predicted Risk of Mortality.

**Table S2.** Procedural characteristics and Complications in the Matched Population.

|  | **S3UR**  **(n=312)** | **S3/S3U**  **(n=312)** | **SMD** |
| --- | --- | --- | --- |
| Valve size |  |  | 0.024 |
| 20 mm | 6 (1.9) | 6 (1.9) |  |
| 23 mm | 83 (26.6) | 78 (25.0) |  |
| 26 mm | 122 (39.1) | 138 (44.2) |  |
| 29 mm | 97 (31.1) | 86 (27.6) |  |
| Approach |  |  | 0.012 |
| Trans-femoral | 299 (95.8) | 300 (96.2) |  |
| Trans-carotid | 7 (2.2) | 3 (1.0) |  |
| Trans-subclavian | 2 (0.6) | 5 (1.6) |  |
| Pre-dilatation | 41 (13.1) | 43 (13.8) | 0.062 |
| Post-dilatation | 31 (10.2) | 35 (11.5) | 0.084 |
| Contrast dye, ml | 80.0 ± 37.0 | 76.5 ± 37.2 | 0.123 |
| Fluoroscopy time, min | 19.5 ± 31.1 | 17.2 ± 14.6 | 0.134 |
| Procedural complications | | | |
| Valve dislocation/embolization | 0 (0) | 0 (0) | - |
| Annulus rupture/aortic dissection | 1 (1.0) | 3 (1.0) | 0.008 |
| Coronary artery occlusion | 2 (0.6) | 1 (0.3) | 0.046 |
| Major vascular complication | 5 (1.6) | 6 (1.9) | 0.084 |

Values are n (%) or mean ± SD. EOA, effective orifice area; IQR, inter-quartile range; PPM, prosthesis-patient mismatch.

**Table S3.** Post-procedural CT analysis in the Sub-group Population

|  | **S3UR**  **(n=105)** | **S3/S3U**  **(n=175)** | **p Value** |
| --- | --- | --- | --- |
| Postimplant oversizing (%) | 4.9 ± 11.8 | 8.4 ± 11.2 | 0.158 |
| Expansion (%) |  |  |  |
| Leaflet outflow | 100.1 ± 7.2 | 102.4 ± 10.5 | 0.224 |
| Prosthesis waist | 97.2 ± 8.8 | 100.2 ± 10.7 | 0.156 |
| Leaflet inflow | 98.8 ± 7.4 | 97.6 ± 7.7 | 0.502 |
| Native annulus | 95.9 ± 8.6 | 97.6 ± 11.2 | 0.423 |
| Eccentricity |  |  |  |
| Leaflet outflow | 0.24 ± 0.09 | 0.25 ± 0.12 | 0.540 |
| Prosthesis waist | 0.30 ± 0.09 | 0.28 ± 0.11 | 0.134 |
| Leaflet inflow | 0.25 ± 0.10 | 0.26 ± 0.11 | 0.645 |
| Native annulus | 0.30 ± 0.11 | 0.27 ± 0.13 | 0.211 |
| Prosthesis deformation index | 1.03 ± 0.07 | 1.02 ± 0.06 | 0.301 |
| HALT | 6 (5.7) | 28 (16.0) | 0.006 |
| HAM | 4 (3.8) | 13 (7.6) | 0.098 |

Values are n (%) or mean ± SD. CT, Computed Tomography; HALT, Hypoattenuated Leaflet Thickening; HAM, Hypoattenuation.

**Table S4.** Predictors of hypoattenuated leaflet thickening (HALT)

| **Variables** | **Univariate** | | **Multivariate** | |
| --- | --- | --- | --- | --- |
|  | **OR (95%CI)** | **p-value** | **OR (95%CI)** | **p-value** |
| Age | 1.00 (0.97-1.03) | 0.950 | 1.05 (0.97-1.13) | 0.219 |
| BMI (per 1) | 1.06 (1.00-1.12) | 0.042 | 1.02 (0.87-1.21) | 0.782 |
| Atrial fibrillation | 0.38 (0.14-1.02) | 0.054 | 1.23 (0.26-5.75) | 0.793 |
| CKD (eGFR<30) | 0.28 (0.04-2.18) | 0.225 |  |  |
| NYHA functional class, III or IV | 1.01 (0.51-2.01) | 0.973 |  |  |
| LVEF (per 1) | 1.01 (0.98-1.03) | 0.732 | 1.01 (0.95-1.07) | 0.713 |
| Aspirin | 1.73 (0.85-3.53) | 0.131 |  |  |
| P2Y12 inhibitor | 0.48 (0.14-1.65) | 0.244 |  |  |
| Any oral anticoagulation | 0.56 (0.21-1.51) | 0.253 |  |  |
| Annular calcium grade moderate or greater | 6.07 (1.57-23.49) | 0.009 | 21.33 (1.85-245.5) | 0.014 |
| LVOT calcium grade moderate or greater | 3.73 (0.67-20.94) | 0.134 |  |  |
| STJ calcium grade moderate or greater | 2.41 (0.47-12.46) | 0.295 |  |  |
| Prosthesis deformation index (per 0.05) | 1.31 (0.46-1.27) | 0.296 | 1.33 (0.70-2.53) | 0.385 |
| S3UR vs S3/S3U | 0.23 (0.10-0.55) | <0.001 | 0.11 (0.02-0.57) | 0.009 |

Data are presented as an estimate (95% confidence interval)

BMI, Body Mass Index; CKD, Chronic Kidney Disease; CI, NYHA, New York Heart Association; LVEF, Left Ventricular Ejection Fraction; LVOT, left ventricular outflow tract; STJ, sino-tubular junction; S3, SAPIEN 3; S3U, SAPIEN 3 Ultra; S3UR, SAPIEN 3 Ultra RESILIA

**Figure S1.** Evaluation of hypoattenuated leaflet thickening (HALT), hypoattenuation affecting motion (HAM), and prosthetic valve geometry.


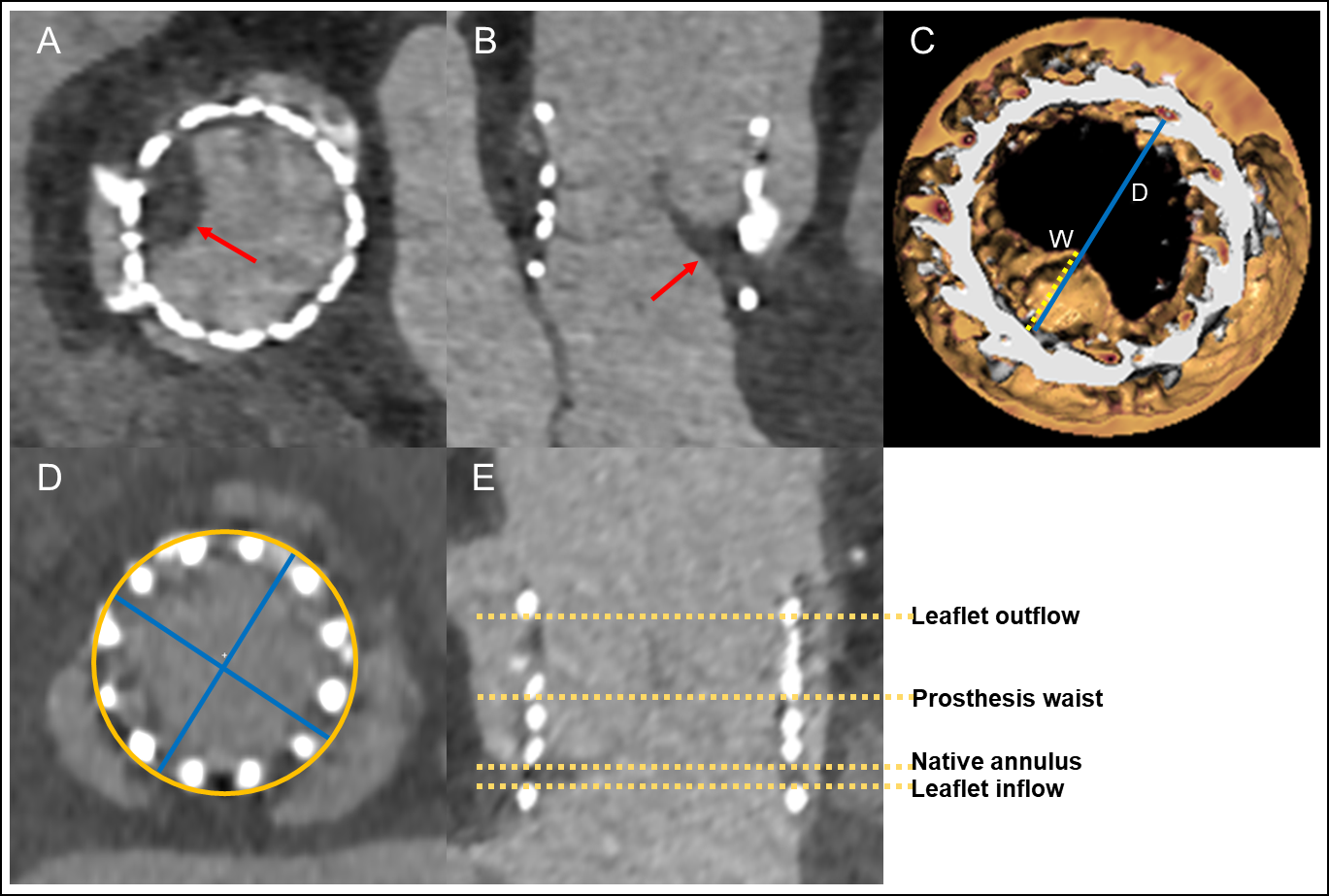


(A, B); We assessed HALT of the valve leaflets using two-dimensional (axial cross-section assessment). HALT was defined as a discernable increase in leaflet thickness during the diastolic phase (**red arrows**). (C); Motion reduction of each leaflet was evaluated using 4D CT volume-rendered en face cine projection in the systolic phase. A quantitative parameter for reduced leaflet motion was calculated as follows: 𝑎=W/(½D) ×100% (**yellow dash line and blue line**). Leaflet motion reduction exceeding 50% of the bioprosthetic frame's radius was classified as HAM. (D, E); A longitudinal view after TAVR. The stent frame area and diameters were measured at specified anatomical levels with yellow dashed lines indicating the levels including, leaflet inflow, native annulus, prosthesis waist, and leaflet outflow.

**Figure S2.** Study flow chart


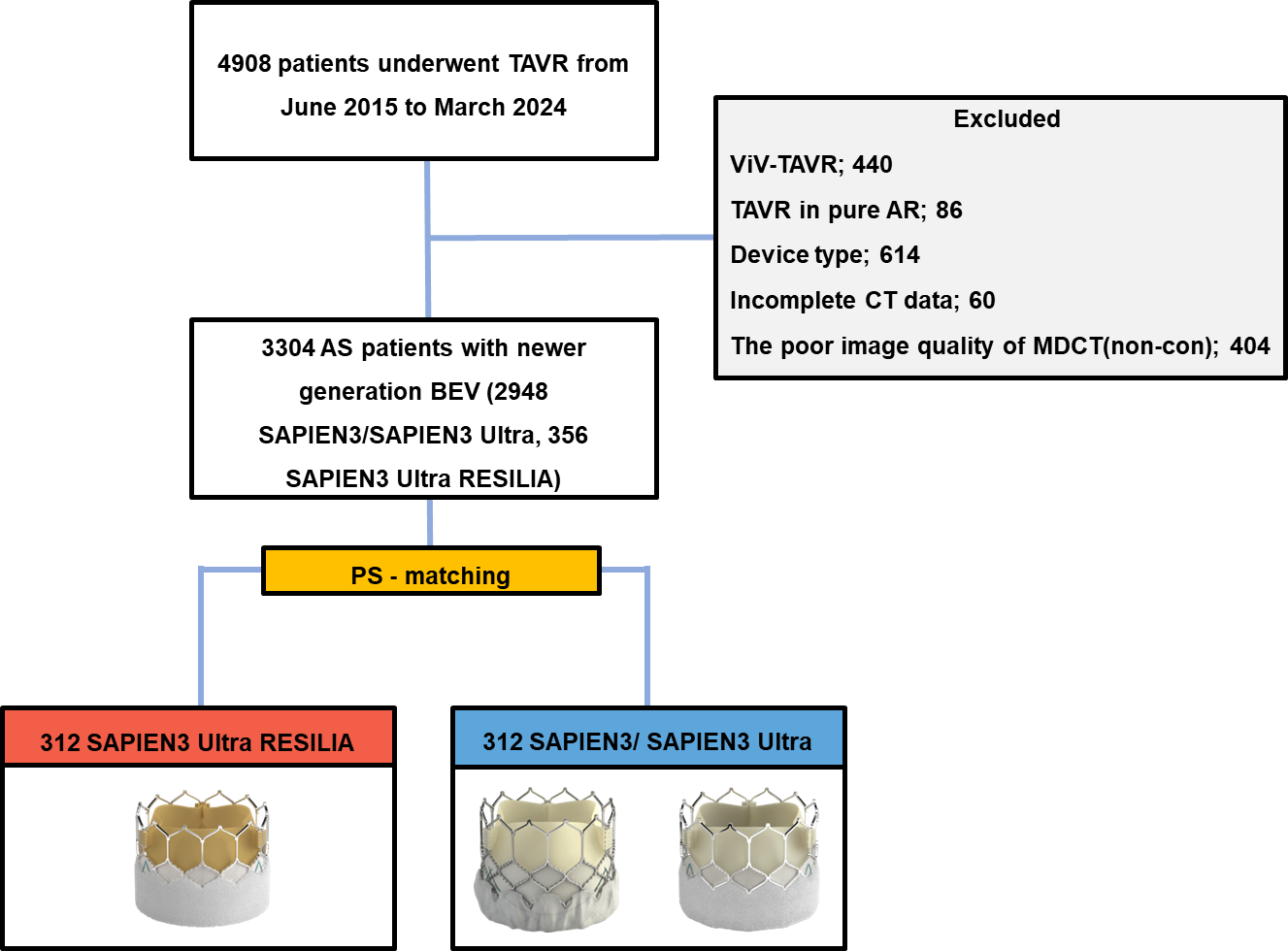


TAVR, transcatheter aortic valve replacement; AR, aortic regurgitation; CT, computed tomography; AS, aortic stenosis; BEV, balloon-expandable valve; PS, propensity score; ViV, valve in valve.

**Figure S3.** Distribution of 30-Day PVL Severity for S3, S3U, and S3UR Valves


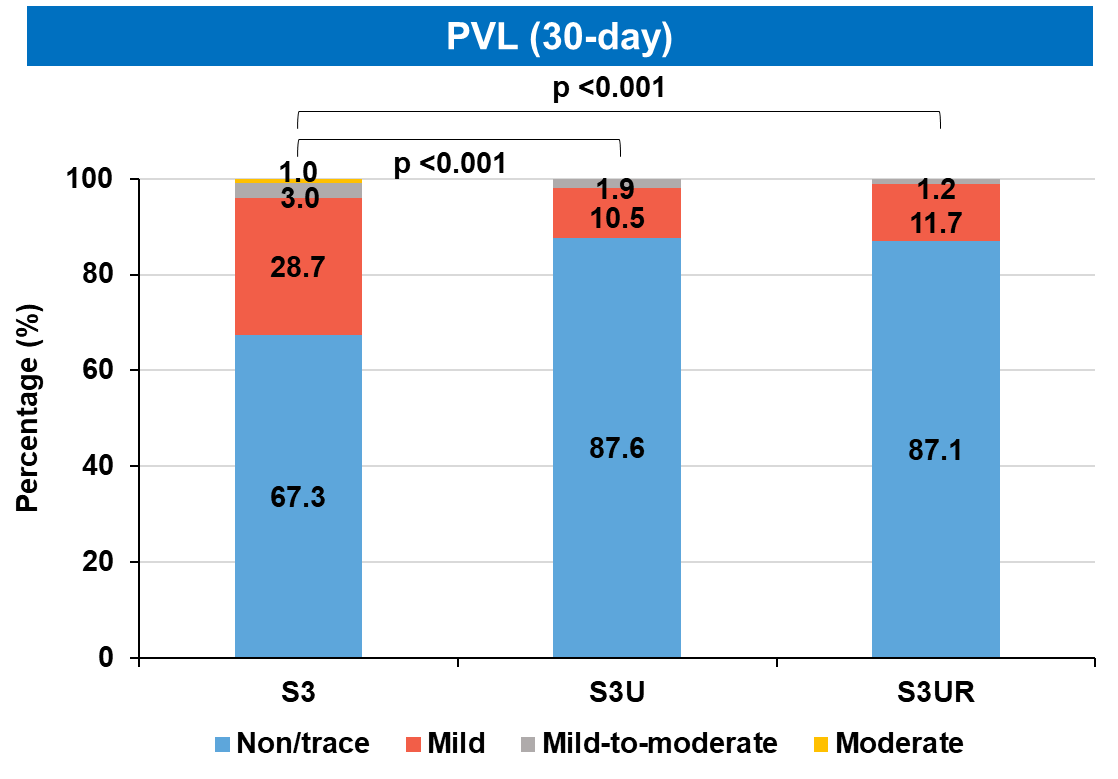


**Figure S4.** Incidence of HALT for S3, S3U, and S3UR Valves


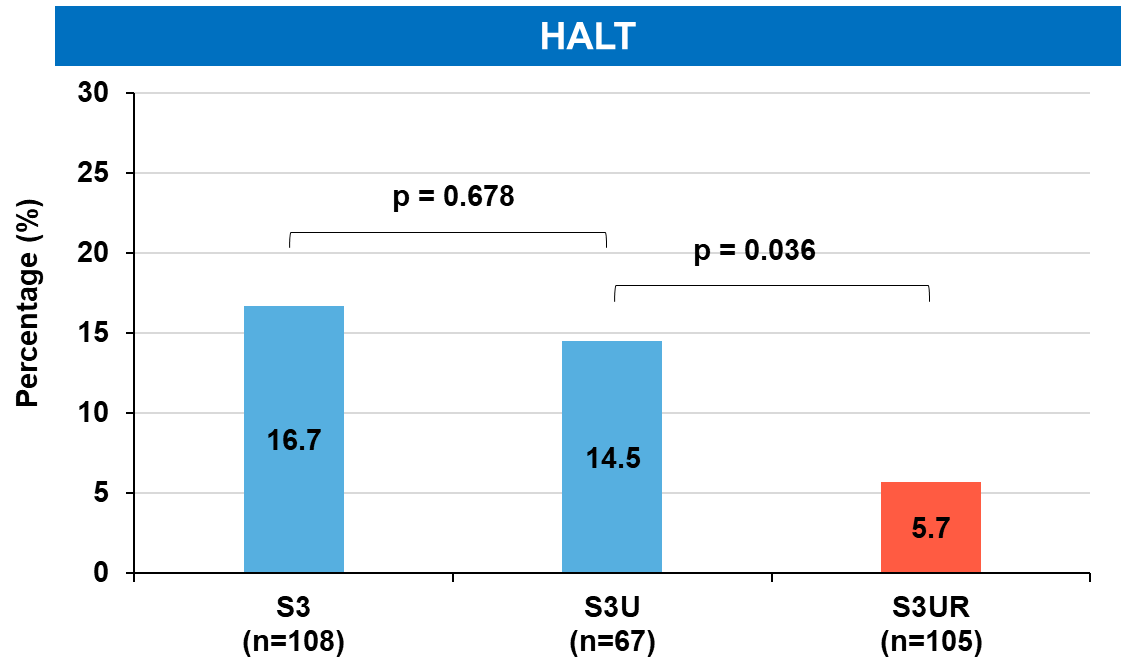

Supplement: Supplementary file 1 — Supplementary file1 (DOCX 1276 KB) [file 12928_2025_1227_MOESM1_ESM.docx]
